# Hallucination or Confabulation? Neuroanatomy as metaphor in Large Language Models

**DOI:** 10.1371/journal.pdig.0000388

**Published:** 2023-11-01

**Authors:** Andrew L. Smith, Felix Greaves, Trishan Panch

**Affiliations:** 1 Department of Psychiatry, University of Ottawa, Ottawa, Ontario, Canada; 2 Department of Mental Health, The Ottawa Hospital, Ottawa, Ontario, Canada; 3 School of Primary Care and Public Health, Imperial College London, London, United Kingdom; 4 Harvard TH Chan School of Public Health, Harvard University, Cambridge, Massachusetts, United States of America; Macquarie University, AUSTRALIA

As Large Language Models (LLMs) and their capabilities become an increasingly prominent aspect of our workflows and our lives, it is important that we are thoughtful and deliberate with the words we use to refer to the inner workings and outputs of this technology. We think that conveying the complex functions (and malfunctions) of LLMs using metaphorical language that is precise and accurate can lead to a better understanding of these powerful tools among both the academic community and the public. If we are meticulous in our choice of metaphors, we open ourselves up to the possibility of achieving a better shared understanding of the complex concepts in this exciting new field. Here, we give the specific example of AI “hallucinations” and demonstrate how a change in metaphorical language can lead to new ways of understanding and may even foreshadow future directions in the development of artificial intelligence systems.

In psychiatry, hallucinations are a relatively well-defined perceptual phenomenon referring to sensory experiences without associated external, or ‘real-world’ stimuli. Clinically, hallucinations are commonly associated with conditions such as schizophrenia, bipolar disorder, and Parkinson’s disease [1]. Employing the term "hallucination" to characterize the inaccurate and non-factual outputs generated by LLMs implies acceptance of the notion that LLMs are engaged in perceiving, that is, becoming consciously aware of a sensory input. While this is a subject of some ongoing debate, there is currently no evidence that AI has gained conscious awareness [2]. LLMs do not have sensory experiences, and thus cannot mistakenly perceive them as real. As such, we believe the term “hallucination” misrepresents the nature of the process occurring within LLMs which it has been used to describe. The model is not “seeing” something that is not there, but it is making things up.

More accurate terminology is found in the psychiatric concept of confabulation, which refers to the generation of narrative details that, while incorrect, are not recognized as such. Unlike hallucinations, confabulations are not perceived experiences but instead mistaken reconstructions of information which are influenced by existing knowledge, experiences, expectations, and context. Confabulation can occur in various clinical conditions including dementia, Wernicke-Korsakoff’s syndrome, schizophrenia, traumatic brain injury (TBI), and cerebrovascular accidents (CVAs) [3]. Confabulation is frequently associated with a generalised lack of awareness of one’s deficits often seen in right sided CVAs or TBIs, as well as in bipolar disorder, schizophrenia, and the dementias [4]. When answering questions, LLMs generate responses based on learned patterns in very large datasets [5]. The output of LLMs can vary significantly due to the probabilistic nature of the transformer architecture [6] and this same lack of deterministic, or rules-based, output likely accounts for the tendency of LLMs to produce non-factual or “confabulated” narrative details. For ease of reference, we provide a summary of the relevant points of comparison between these terms in Table 1.

**Table 1:** "Hallucination" vs "Confabulation" for LLM Output.

Term	Pros	Cons
**Hallucination** *Sensory experiences not associated with an external stimulus*	Commonly understood term Captures the ’creation’ aspect of LLM outputs	Suggests a sensory perception, not applicable to LLMs Implies the presence of consciousness or subjective experience, which LLMs do not have
**Confabulation** *Production of a false memory that is not intended to deceive*	Accurately describes the pattern-based, context-dependent generation of content by LLMs Does not imply consciousness or subjective experience	Less commonly understood term Less evocative

In using language derived from human neurocognitive processes, we recognize the risk of seeming to advance an anthropomorphic view of LLMs [7]. This is not our intention and does not accurately reflect our thinking. Rather than anthropomorphizing LLMs as displaying human traits and behaviours and thereby implying capacity for empathic connection, motivation for power, and other similarly far-fetched ideas, we instead believe that using linguistic terms more faithful to the underlying technology provides needed clarity upon which to build trust and drive adoption. Using metaphoric language with precision, functional aspects of LLMs can be subjected to analogical reasoning, thereby yielding plausible roadmaps for development of more powerful, more advanced, and more helpful models.

As noted above, confabulation is often associated with clinical conditions involving right hemispheric deficits. The right hemisphere of the brain is thought to be responsible for a wide range of cognitive functions, including processing non-verbal cues, understanding the emotional state of others, and appreciating the nuances of music [8]. When this hemisphere is damaged the left hemisphere predominates but does so in a more literal and simplistic way. This can lead to a focus on detail, a preference for sequencing and ordering, and an overly optimistic and often unrealistic assessment of the self [8].

We suggest that the analogy between the left hemisphere’s orientation to the world and current LLMs is instructive. With the availability of massive computational power, LLMs vastly outperform the human brain’s ability to absorb and retain large amounts of information and can produce outputs on a scale that no individual human could. Yet LLMs, like the unmitigated, confabulating left hemisphere, may confidently produce false information. We propose that the “human-in-the-loop” approach [9] to responsible use of AI in the medical context may be seen as a reintroduction of the contextualising and sense-making functions of the right hemisphere, in the form of direct human oversight. While the landscape here changes rapidly, at this time humans remain better suited to real-world decision making under conditions of uncertainty and are for now alone in our capacity for empathy, embodiment, and the complex value-based prioritisation required to make judgements in medical care. In the collaboration between humans and AI technology, we witness a synergistic relationship reminiscent of the brain’s right and left hemispheres. Just as these brain regions possess unique strengths that complement each other, humans and AI each contribute capabilities that may compensate for the other’s limitations. This partnership bears the promise of reshaping industries and solving unimaginably complex problems.

Over the longer term will we see development of artificial intelligence analogues to right hemispheric ways of thinking? We think the answer is in many ways, yes, though perhaps not to the dystopian extent that some may fear. Artificial correlates of empathy may yet be years away, but we see multiple specialised LLMs interacting in a structured way, much like the gated interconnectivity of our neuroanatomy, on the very near-term horizon [9]. There is collective wisdom preserved in evolution’s partitioning of selectively interconnected brain structures, which we believe provides a map for our approach to the development of advanced AI systems.

The use of inaccurate language often leads to pervasive misunderstandings which become increasingly difficult to correct over time. Particularly in the medical context, where adoption of new technologies can have both immediate and long-term implications for the health and well-being of the population, it is important that we choose our words, and thus our metaphors carefully. We propose using the term “confabulation” not merely to correct a misnomer, but because the neuroanatomical analogy it implies unlocks new ways of understanding and suggests exciting new paths for technological advancement.
